# Targeting Insulin-Like Growth Factor 1 Receptor Inhibits Pancreatic Cancer Growth and Metastasis

**DOI:** 10.1371/journal.pone.0097016

**Published:** 2014-05-08

**Authors:** Ramadevi Subramani, Rebecca Lopez-Valdez, Arunkumar Arumugam, Sushmita Nandy, Thiyagarajan Boopalan, Rajkumar Lakshmanaswamy

**Affiliations:** Center of Excellence in Cancer Research, Department of Biomedical Sciences MSB1, Texas Tech University Health Sciences Center, Paul L. Foster School of Medicine, El Paso, Texas, United States of America; Thomas Jefferson University, United States of America

## Abstract

Pancreatic cancer is one of the most lethal cancers. Increasing incidence and mortality indicates that there is still much lacking in detection and management of the disease. This is partly due to a lack of specific symptoms during early stages of the disease. Several growth factor receptors have been associated with pancreatic cancer. Here, we have investigated if an RNA interference approach targeted to IGF-IR could be effective and efficient against pancreatic cancer growth and metastasis. For that, we evaluated the effects of IGF-1R inhibition using small interfering RNA (siRNAs) on tumor growth and metastasis in HPAC and PANC-1 pancreatic cancer cell lines. We found that silencing IGF-1R inhibits pancreatic cancer growth and metastasis by blocking key signaling pathways such AKT/PI3K, MAPK, JAK/STAT and EMT. Silencing IGF-1R resulted in an anti-proliferative effect in PANC-1 and HPAC pancreatic cancer cell lines. Matrigel invasion, transwell migration and wound healing assays also revealed a role for IGF-1R in metastatic properties of pancreatic cancer. These results were further confirmed using Western blotting analysis of key intermediates involved in proliferation, epithelial mesenchymal transition, migration, and invasion. In addition, soft agar assays showed that silencing IGF-1R also blocks the colony forming capabilities of pancreatic cancer cells *in vitro.* Western blots, as well as, flow cytometric analysis revealed the induction of apoptosis in IGF-1R silenced cells. Interestingly, silencing IGF-1R also suppressed the expression of insulin receptor β. All these effects together significantly control pancreatic cancer cell growth and metastasis. To conclude, our results demonstrate the significance of IGF-1R in pancreatic cancer.

## Introduction

Pancreatic cancer is the fourth leading cause of cancer related death even though it is only the thirteenth most common malignancy in the world [1]. The 5 year survival rate for patients with pancreatic cancer is the lowest reported for any cancer, which is less than 1% [2]. This is mainly because pancreatic cancer is difficult to detect at early stages due to lack of early warning signs or symptoms [1]. Pancreatic ductal adenocarcinoma (PDAC), which is the most common form of this extremely aggressive cancer, is highly invasive and metastatic and is highly resistant to all forms of existing therapies [3]. Patients who are diagnosed with PDAC at advanced stages have little hope of effective surgical resection [1] and other treatments like radiation, chemotherapy (Gemcitabine, 5-flurouracil, cisplatin, paclitaxel, docetaxel, etc.) or targeted therapies (Erlotinib-Tarceva) [4] do not currently offer much benefit either. The asymptomatic nature of the disease in its early stages has ensured that PDAC still remains a deadly and nearly untreatable cancer despite multiple attempts to find better treatment strategies [5]. According to the most recent report, approximately 280,000 new pancreatic cancer cases are diagnosed globally and the incidence of pancreatic cancer continues to increase [6], [7]. Increasing incidence and mortality indicates that there is still much lacking in detection and management of the disease. Therefore, it is absolutely necessary to find better diagnostic and therapeutic strategies for treating this disease.

Several growth factor receptors such as insulin–like growth factor 1 receptor (IGF-1R), epidermal growth factor receptor (EGFR), etc., are aberrantly expressed in many types of cancer including pancreatic cancer [8]. Increased IGF-1R expression levels are associated with higher risk of developing various neoplasms [9]. The IGF-1R signaling axis is highly activated during the early stages of lung carcinogenesis, where a role for IGF-1R signaling was demonstrated not only in primary tumor formation but also in progression to more aggressive lung adenocarcinoma [10]. Jie Tang et al., 2013 reported high expression levels of IGF-1R in tumor tissue samples from 25 of 36 patients with epithelial ovarian cancer. They also reported consistently higher levels of IGF-1 in primary cancer cell cultures (230 ng/ml) compared to normal ovarian tissue cell cultures (101.9 ng/ml) [11]. IGF-1R signaling activates intracellular signaling cascades that include phosphatidyl inositol 3-kinase (PI3K), AKT, Rac and mitogen-activated protein kinase (MAPK) [12], [13]. These pathways regulate key genes involved in various cellular functions such as proliferation, survival, differentiation, transformation and apoptosis [10]. Moreover, IGF-1R targets 70 to 100% of the core metabolic pathways that are often altered in PDAC pathogenesis [14]. Targeting IGF-1R has already been shown to enhance the therapeutic effects of mTOR inhibitors in metastatic renal cell carcinoma, [15]. Likewise, in human epithelial ovarian cancer, targeting IGF-1R by antisense nucleotide reduced proliferation by 70% and clonogenicity by 10 fold [11]. In both in vitro and in vivo systems, an IGF-1R antagonist (monoclonal antibody MK-0646) was shown to significantly down regulate X-linked inhibitor of apoptosis (XIAP) protein, which has been shown to be involved in cell survival and inhibition of cell death in colorectal cancer [16]. Moreover, IGF-1R targeted therapies have already moved forward into phase I clinical trials for prostate, breast, colorectal, liver, synovial sarcoma, etc., [12], [17]–[19] with promising initial results. However, the potential benefit of using IGF-1R targeted therapy in pancreatic cancer is not fully explored. Further, there is still a lack of detailed knowledge regarding the exact molecular mechanisms by which IGF-1R regulates pancreatic carcinogenesis.

Therefore, based on mounting evidence for the efficacy of targeting IGF-1R in a broad spectrum of cancers, we have determined the effects of targeting IGF-1R in pancreatic cancer in this study. The ultimate goal of this study is to identify whether IGF-1R signaling is an effective therapeutic target for pancreatic cancer with the potential to translate rapidly into clinical use. Here we clearly demonstrate that blocking IGF-1R expression enhances apoptosis and suppresses cell invasion, migration and metastasis via modulation of PI3K/AKT, MAPK and JAK/STAT signaling pathways in pancreatic cancer cells.

## Materials and Methods

### Ethics Statement

All the experiments performed were approved by and performed following the guidelines of the Institutional Biosafety Committee of Texas Tech University Health Sciences Center.

### Cell Lines and Reagents

Human pancreatic ductal adenocarcinoma cell lines, PANC-1 (epithelioid carcinoma), MIA PaCa-2 (carcinoma) and HPAC (adenocarcinoma) were purchased from the American Type Culture Collection, and were maintained in RPMI-1640 medium supplemented with 10% FBS, 100 Units/mL of penicillin, and 100 µg/mL of streptomycin. Cells were maintained at 37°C in a humidified atmosphere containing 5% carbon dioxide.

TransIT- siQUEST transfection reagent was purchased from Mirus Bio (Madison, WI, USA). The 6.5 mm Transwell with 8.0 µm pore polycarbonate membrane inserts was obtained from Corning Incorporated (Corning, NY, USA). BD Matrigel (Bedford, MA, USA) and BD Pharmingen Annexin V-FITC Apoptosis Detection Kit I (San Diego, CA, USA) was obtained from BD Biosciences. BSA was purchased from Sigma-Aldrich Corporation (St Louis, MO, USA). siRNA targeting IGF-1R was purchased from Origene (Rockville, MD,USA). MTS reagent [3-(4, 5-dimethylthiazol-2-yl)-5-(3-carboxymethoxyphenyl)-2-(4-sulfophenyl)-2H-tetrazolium] was obtained from Promega (Madison, WI, USA). Mammalian protein extraction reagent (mPER) was purchased from Thermo Scientific (Rockford, IL, USA).

The following primary antibodies were used in this study: pAKT(sc-101629), AKT(5298), Bcl-2(sc-783), pERK, (sc-101760), ERK (sc-94) and STAT3(H-190)(sc-7179) (Santa Cruz,CA,USA); IGF-1R(3027), Notch 2 (4530P), Snail (3879), E-cadherin (3195), N-cadherin (4061), Zeb (3396), Vimentin (5741), Slug (9585), Bax (2772), Caspase3 (9661), PARP (9542), pPI3K p85(4228), PI3K p85(4292), IR-β (3024), pIRS-1(2388), IRS-1(2382), pSTAT3 (ser727) (4113), COX-2 (4842), pPTEN (9549), pmTOR (2974), mTOR(4517), p-p70s6kinase (9206) and p70s6kinase (9202) (Cell Signaling Technology, (Boston, MA); Caspase8 (ab 25901) (Abcam, Cambridge, MA, USA); β-actin (Sigma Aldrich, (St.Louis, MO, USA). Appropriate secondary antibodies were obtained from Santa Cruz Biotechnology (Santa Cruz, CA, USA).

### Pancreas Adenocarcinoma Tissue Array

Pancreas adenocarcinoma tissue microarray (TMA) was obtained from US Biomax, Inc, Rockville, MD The TMA containing formalin-fixed paraffin embedded samples of pancreatic adenocarcinoma and normal pancreatic tissues was subjected to immunohistochemistry (IHC) to determine IGF-1R expression levels. Institutional Review Board approval was not required for using TMAs.

### Immunohistochemistry (IHC)

IHC for IGF-1R antigen was performed using the pancreas adenocarcionma TMA. TMAs were first incubated in an oven at 58°C for 2 h to enhance tissue adhesion to the charged glass slides. Deparaffinization was then carried out to remove embedded medium using xylene incubation for 20-minute. TMAs were gradually rehydrated in serial alcohol baths (100, 95, 70, 50 and 30%) followed by a distilled water wash for 5 min. Heat induced epitope retrieval with trilogy (Cell Marque, Rocklin, CA) was then performed to unmask the antigenic sites within the tissue sections. TMAs were blocked in TBS containing 1% fetal calf serum and 1% bovine serum albumin for 15 min. Perox-free blocking reagent (Cell Marque, Rocklin, CA) was also added for 10 min to block non-specific antibody binding. TMAs were incubated with IGF-1R antibody (1∶50 dilution) overnight at 4°C. Slides were then washed three times in PBS for 5 min and incubated with Ultra Marque polyscan HRP Label (Cell Marque, Rocklin, CA) for 1 h at room temperature. TMAs were then washed three times in PBS and stained with chromogen solution (Cell Marque, Rocklin, CA) for 20 min. Chromogen staining reaction was stopped by rinsing with distilled water. Cell nuclei counterstaining was performed with hematoxylin incubation for 40 sec. TMAs were rinsed with distilled water and dehydrated with serial ethanol baths (30, 50, 70, 95 and 100%) followed by a xylene bath. Finally, TMAs were coverslipped with the mounting media (Surgipath Medical Industries, Richmond, IL) and digital images were captured using a Nikon Microscope- ECLIPSE 50i.

### Silencing of IGF-1R in PANC-1 and HPAC Cells

siRNA targeting IGF-1R were transiently transfected into PANC-1 and HPAC cells using MIrus bio TransIT siQUEST transfection reagent. Scrambled siRNA (non-silencing sequence) was used as a control. Briefly, cells were seeded in 6-well plates at a density of 2.5×10^5^ cells/well. Cells were transfected with different concentrations and different subtypes (A, B and C) of siRNA ranging from 10 to 50 nM for 48 h or 72 h, using Mirus siQUEST Transfection Reagent. The ratio of siRNA to Transfection reagent was maintained as 1∶0.5 for efficient silencing without toxicity according to the manufacturer’s protocol. The final concentrations of siRNA were chosen based on dose–response studies. Forty-eight hours after the transfection, cells were used for protein isolation or clonogenicity, invasion, and migration, studies. Apoptosis was studied at 48 and 72 hours after silencing of IGF-1R.

### Cell Viability Assay

PANC-1 and HPAC cells were seeded in 96-well plates at a density of 0.3×10^4^ cells/well and 0.5×10^4^ cells/well, respectively, and transfected with IGF-1R at a final concentration of 30 nM of subtype “B” and 50 nM of subtype “A” for 48 h along with scrambled controls. After 48 h of transfection, cell viability was measured using the MTS assay. The absorbance was read at 490 nm in order to calculate the percentage viable cells.

### Clonogenicity/Colony Formation Assay in Soft Agar

Colony formation assay was performed in order to measure the in vitro survival ability of a single cell to grow into a colony in an anchorage–independent growth environment. Briefly, after transfecting PANC-1 and HPAC cells with IGF-1R siRNA or scrambled control for 48 h, these transfected cells were seeded in complete media at a density of 2×10^4^ cells in 60-mm dishes containing a top layer of 0.7% agar and a bottom layer of 1% agar. The plates were incubated at 37°C for 3 to 4 weeks and then stained with 0.2% Crystal violet. Colonies of greater than 20 cells were counted manually.

### Wound Healing Assay

Cell migrating ability of IGF-1R silenced pancreatic cancer cells were measured using the scratch assay. Briefly, cells were seeded in 6-well plates at a density of 3.5×10^5^ cells/well for IGF-1R transfection. At this density, PANC-1 and HPAC cells reached monolayer confluency after 48×h. A straight wound or scratch was then gently created in the cell monolayers with a sterile pipette tip. Cells detached by the scratch were washed twice with PBS and cultures were then supplemented with fresh medium and monitored for 96 h at 37°C using the Biostation CT (Nikon Instruments Inc. Melville, NY, USA) for continuous observation. The Biostation was automatically programmed to capture photographs at 2 h intervals up to 96 h. Migration images were captured and documented at different time points using NIS-Element AR software.

### Migration and Invasion Assay

The effect of IGF-1R siRNA on invasive properties of pancreatic cancer cells was evaluated using transwell migration and invasion assays. Forty-eight hours after transfection, PANC-1 cells and HPAC cells were trypsinized and resuspended in FBS-free RPMI-1640 medium. For the migration assay, a total of 5×10^3^ cells were plated in the top chamber of the transwell with a noncoated polycarbonate membrane (6.5 mm diameter insert, 8.0 ìm pore size; Corning Incorporated). For the invasion assay, 2×10^4^ cells were plated in the top chamber of the transwell with a matrigel-coated (1 mg/mL) polycarbonate membrane. RPMI-1640 medium with 10% FBS was added to the lower chamber as a chemoattractant. After incubation for 48 h at 37°C with 5% CO_2_, cells on the lower surface of the membrane were fixed with 5% formalin and stained with 0.2% crystal violet. The non-migrated cells on the upper side of the insert were wiped off with a cotton swab. Images of the cells which migrated to the undersurface of the membrane were captured in a blinded manner at 5 different microscopic fields with 20× magnification. The number of migrated or invaded cells was counted from five or six randomly selected fields in a blinded manner. Experiments were done in triplicates for statistical significance.

### Analysis of Cell Death

The effect of IGF-1R silencing on apoptosis was measured using the Annexin V-FITC Apoptosis Detection Kit I. Cells were harvested 48 h and 72 h post-transfection and then stained with Annexin V-FITC and propidium iodide according to the manufacturer’s instructions. The percentage of cell death or apoptosis was quantified using a flow cytometer (FACS Accuri C6) [20].

### Western Blotting Analysis

PANC-1 and HPAC cells were transfected with IGF-1R siRNA for 48 h. After the incubation, protein was extracted from transfected cells by lysing the cell membrane using mammalian Protein Extraction Reagent (mPER) according to the manufacturer’s protocol. The protein concentration was quantitated using BSA standard methodology. Equal amounts of protein were loaded and separated by SDS-polyacrylamide gels, and transferred onto PVDF membranes. The membranes were blocked with 5% BSA in 1 X TBST for 1 h and probed with a panel of primary antibodies against pAKT, AKT, Bcl-2, pERK, ERK, STAT3, IGF-1R, Notch 2, Snail, E-cadherin, N-cadherin, Zeb, Vimentin, Slug, Bax, Caspase3, PARP, pPI3K p85, PI3K p85, IR-β, pIRS-1, IRS-1, pSTAT3, COX-2, pPTEN, pmTOR, mTOR, pp70s6kinase, p70s6kinase, Caspase8 or β-actin. After washing with TBS-T, the membrane was incubated with secondary horseradish peroxidase-coupled antibodies and then positive bands were visualized using enhanced chemiluminescence.

### Statistical Analysis

The differences between groups were analyzed by unpaired student’s t-test. The GraphPad Prism 5 software package version 5.03 was used to do all the statistical calculations. Probability values <0.05 were considered to be statistically significant.

## Results

### IGF-1R is Highly Expressed in Pancreatic Cancers

**Figure 1:**
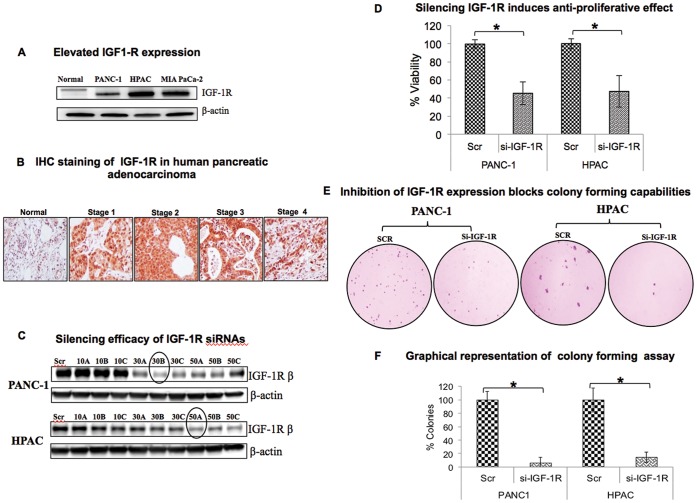
Effect of silencing IGF-1R on proliferation and colony formation in pancreatic cancer cell lines. (A) Expression levels of IGF-1R in PANC-1, HPAC and MIA PaCa-2 were compared with normal pancreatic cells from rat using western blot. (B) Representative immunohistochemical analysis of IGF-1R by tumor stage in pancreatic adenocarcinoma tissues and normal pancreas tissue. (C) PANC-1 and HPAC cells were transfected with three predesigned IGF-1R siRNAs (A, B and C) at three different concentrations (10, 30 and 50 nM) along with scrambled control siRNA. Silencing efficacy of IGF-1R siRNA was determined using western blot in PANC-1 and HPAC cells. (D) Effect of IGF-1R siRNA on cell viability of PANC-1 and HPAC. Cells were transfected with 30 nM and 50 nM of IGF-1R siRNA in PANC-1 and HPAC cells respectively. Cell viability was assayed at 48 h post transfection using MTS assay kit. Results represented as mean ± standard deviation (n = 3). (E) Inhibition of IGF1R expression blocks colony forming capabilities of pancreatic cancer cells, PANC-1 and HPAC. A soft agar assay was used to study the colony formation ability of PANC-1 and HPAC cells. Forty eight hours after the siRNA transfection, PANC-1 and HPAC cells were allowed to grow in 0.7% agarose in RPMI-1640-supplemented with 10% FBS for 16 and 22 days, respectively. Shown here are representative pictures of colony formation from two independent experiments done in triplicate. (F) Percentage colonies in both PANC-1 and HPAC cells were calculated with scrambled control (SCR) serving as the baseline.

We examined the expression levels of IGF-1R in pancreatic ductal adenocarcinoma cell lines (HPAC, MIA PaCa-2 and PANC-1) using western blot. All three cell lines had a high level of expression of IGF-1R compared to normal pancreas. HPAC had the highest expression of IGF-1R among the three pancreatic cell lines we analyzed while PANC1 had the least expression. So we chose to use HPAC and PANC1 cells for all our experiments based on their IGF-1R expression (Figure 1A). Immunohistochemical analysis of IGF-1R in pancreas adenocarcinoma tissue microarray (TMA) was done to further confirm the pathophysiological role of IGF-1R in PDAC pathogenesis. PDAC tumor tissues at various stages showed increased expression of IGF-1R compared with normal pancreas tissues (Figure 1B).

### Silencing IGF-1R using siRNAs in Pancreatic Cancer Cell Lines

To silence IGF-1R expression, we used 3 different siRNAs at concentrations ranging from 10 to 50 nM. Scrambled siRNA which does not target any gene was used as a control. siRNA transfection efficiency varied based on the subtype and concentration of siRNA for both cell lines. Accordingly, IGF-1R expression levels were significantly reduced at a concentration of 30 nM “B” and 50 nM “A” for PANC-1 and HPAC cancer cells, respectively (Figure 1C). Therefore, these two siRNA doses were selected for all subsequent studies.

### Silencing IGF-1R Induces Anti-proliferative Effect in Pancreatic Cancer Cell Lines

Since IGF-1R is known to stimulate cell proliferation, we examined the effect of silencing IGF-1R on proliferation of pancreatic adenocarcinoma cells. Silencing IGF-1R decreased the viability of pancreatic cancer cells at 48 h post-transfection compared with scrambled siRNA transfected control cells. Only 45.24% & 47.28% cells were viable upon IGF-1R inhibition in PANC1 and HPAC cells, respectively (Figure 1D). The anti-proliferative effect of targeting IGF-1R is highly significant in both of the highly aggressive pancreatic cancer cell lines. These results suggest a pivotal role for IGF-1R in the proliferation of aggressive pancreatic cancer cells.

### Silencing IGF-1R Inhibits Anchorage-independent Growth of Pancreatic Cancer Cells

Anchorage-independent growth potential of cancer cells is one of the important and well-known characteristic features of transformed cells. Soft agar assays in PANC-1 and HPAC cells were performed to assess whether IGF-1R knockdown influenced colony forming potential of these cells. Silencing IGF-1R dramatically reduced the colony forming capacity in both pancreatic cancer cell lines (Figure 1E). Results from three independent experiments were quantified, which reveals >85% inhibition of colony forming capability in IGF-1R silenced pancreatic cancer cells (Figure 1F).

### IGF-1R Silencing Suppressed Pancreatic Cancer Cell Migration/motility

#### (i) Wound healing assay

**Figure 2:**
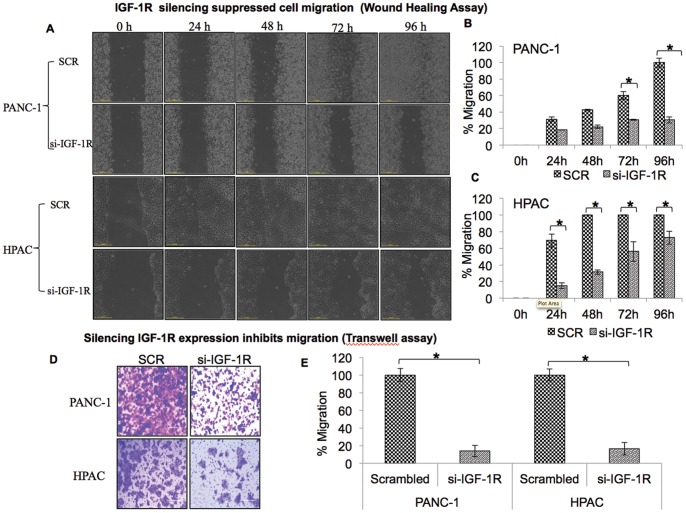
IGF1R silencing suppressed cell migration in pancreatic cancer cell lines. (A) Wound healing assay was performed to evaluate the migration of PANC-1 and HPAC cells after silencing IGF-1R. Forty eight hours after siRNA transfection, wound healing capacity of cells were monitored with automated Nikon Biostation CT at 2 h intervals up to 96 h. (B & C): Cell migration was determined by the rate of cells moving towards the scratched area. The percentage migration was calculated by the NIS-Element AR software. Similar results were obtained in three independent experiments. (D) Silencing IGF-1R expression inhibits migration of PANC-1 and HPAC cells. Cell migratory abilities were determined using uncoated transwell Boyden chambers. Post transfection PANC-1 and HPAC cells were allowed to migrate through pores to the bottom surface of transwell. Migrated cells were fixed and stained with 0.2% crystal violet in 5% formalin. Data are representative of five random microscopic field images taken at 20X magnification (E) Percentage migration for transwell assays is shown for IGF-1R silenced PANC-1 and HPAC cells from the results of three independent experiments.

Reduced colony forming ability is generally associated with a corresponding loss of invading capabilities of cancer cells [20]. The classic wound healing assay was performed to test the role of IGF-1R in regulating the migratory ability of pancreatic cancer cells. Cell monolayers were scratched to create a wound to monitor the migrating ability of both control and IGF-1R suppressed pancreatic cancer cells. IGF-1R suppressed PANC-1 and HPAC cells exhibited reduced migratory capabilities compared with scrambled controls. HPAC scrambled control cells reformed a complete monolayer within 48 h and PANC-1 scrambled control cells reformed a complete monolayer within 96 h. In IGF-1R suppressed PANC-1 and HPAC cells, a complete monolayer was not reformed even after 96 h (Figure 2A, B & C).

#### (ii) Transwell migration assay

Suppressed migration of PANC-1 and HPAC cells achieved by blocking IGF-1R expression was further confirmed using transwell migration assays. Once again, compared to controls, IGF-1R siRNA transfected PANC-1 and HPAC cells showed a significant decrease in the number of cells that migrated (Figure 2D & E). This strongly indicates that blocking IGF-1R expression suppresses the migrating abilities of both aggressive pancreatic cancer cell lines.

### Knockdown of IGF-1R Inhibits Cell Invasion

**Figure 3:**
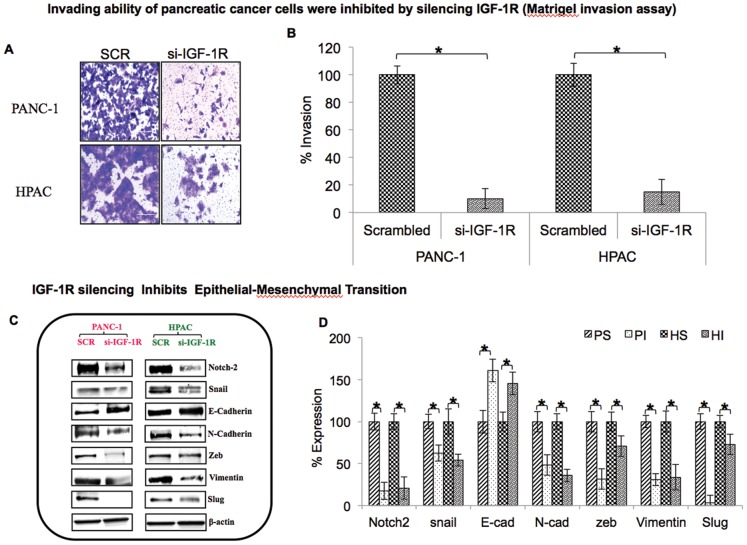
IGF-1R silencing inhibits invading ability and epithelial-mesenchymal transition of pancreatic cancer cells. (A) PANC-1 and HPAC cell invasion was assessed in transwell chambers coated with matrigel. Cells that invaded the matrigel-coated insert were fixed, stained and captured at 20× magnification. (B) Number of invaded cells were counted and expressed as percentage invasion. Experiments were done in triplicate (C) IGF-1R silencing inhibits expression of several epithelial-mesenchymal transition markers. Total protein lysates from scrambled control and IGF-1R silenced PANC-1 and HPAC cells were analyzed for expression of Notch-2, Snail, E-cadherin, N-Cadherin, Zeb, Vimentin, and Slug along with internal control β-actin. (D) Densitometic values of EMT markers are shown as % expression. PS-PANC-1 Scrambled, PI-PANC-1 IGF-1R silenced, HS-HPAC Scrambled, HI-HPAC IGF-1R silenced.

Cell invasion is one of the critical steps involved in cancer cell metastasis. Escape of cancer cells from primary site of origin to distant sites occurs when the cells acquire the ability to penetrate the tumor basement membrane and invade into surrounding tissue and eventually into the blood and lymphatic systems. In vitro matrigel invasion assays using transwell Boyden chambers which mimic the internal basement membrane of the tumor microenvironment were used to investigate the invading potential of IGF-1R siRNA transfected pancreatic cancer cells. Consistent with reduced migratory ability, cells treated with IGF-1R siRNA demonstrated a significant reduction in cell invasion ability of more than 85% in PANC-1and HPAC cells compared with scrambled siRNA-treated cells (Figure 3A & B). Taken together, these results indicate that silencing IGF-1R not only decreases migration ability, but also the invasive properties of pancreatic ductal adenocarcinoma cells.

### Silencing IGF-1R Blocks Epithelial-mesenchymal Transition (EMT) in Pancreatic Cancer Cells

The process of cancer cell invasion is enabled by EMT which is the initiator of the metastatic cascade [21]. Cells which undergo EMT will attain stem cell-like properties that increase cell proliferation, metastasis, etc. [22]. The EMT-related factors such as Notch-2, Snail, N-cadherin, Zeb, Vimentin and Slug were significantly reduced upon silencing IGF-1R in PANC-1 and HPAC cells (Figure 3C & D). Interestingly, western blot analysis in IGF-1R suppressed cells showed increased expression of E-cadherin; loss of this cell-cell adhesion molecule is thought to promote invasion and metastasis [23]. These data clearly indicate that silencing IGF-1R in pancreatic cancer cells results in inhibition of proteins favoring pancreatic cancer EMT.

### Silencing IGF-1R Induces Apoptosis

**Figure 4:**
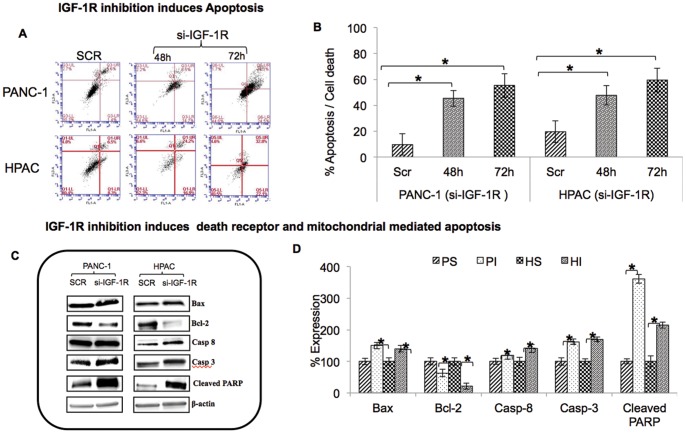
The effect of IGF-1R siRNA on apoptosis of pancreatic cancer cells. (A) IGF1R inhibition induces apoptosis in PANC1 & HPAC cells. Post transfection cells were stained with Annexin-V-FITC and propidium iodide followed by flow cytometry. The percentage of early apoptotic (bottom right quadrant), apoptotic (top right quadrant), late apoptotic and necrotic cells (top left quadrant), and live healthy cells (bottom left quadrant) are shown. (B) Percentage apoptosis and cell death is summarized for three independent experiments in PANC-1 and HPAC cells. (C) IGF-1R inhibition induces death receptor and mitochondrial mediated apoptosis in PANC1 & HPAC cells. Bax, Bcl-2, caspase 8, caspase 3 and cleaved PARP and β-actin expression was assayed using Western blot in IGF-1R siRNA-transfected PANC-1 and HPAC cells. (D) Representative densitometry analysis shows significant potentiation of apoptosis via intrinsic and extrinsic pathways. PS-PANC-1 Scrambled, PI-PANC-1 IGF-1R silenced, HS-HPAC Scrambled, HI-HPAC IGF-1R silenced.

Regulation of apoptosis in pancreatic cancer cells was analysed upon IGF-1R silencing using Annexin V-FITC Apoptosis Detection Kit I. Flow cytometric analysis showed a pronounced induction of apoptosis/cell death by IGF-1R silencing in pancreatic cancer cells (45.4%, 55.4% in PANC-1 and 47.7%, 59.5% in HPAC was observed after 48 h and 72 h of post transfection, respectively) (Figure 4A & B).

To identify the molecular mechanism involved in this induction of apoptosis, we investigated the expression pattern of several apoptotic signaling molecules in pancreatic cancer cells. Cells transfected with IGF-1R siRNA had significantly increased expression of Bax, Caspase 8, Caspase3 and cleaved PARP in comparison with cells treated with scrambled siRNA (Figure 4C & D). Silencing IGF-1R also decreased the anti-apoptotic protein Bcl-2 in both PANC-1 and HPAC cells (Figure 4C & D). These data reveal that IGF-1R siRNA induces apoptosis via both death receptor and mitochondrial mediated pathways of apoptosis.

### Silencing IGF-1R Alters Key Signaling Molecules

**Figure 5:**
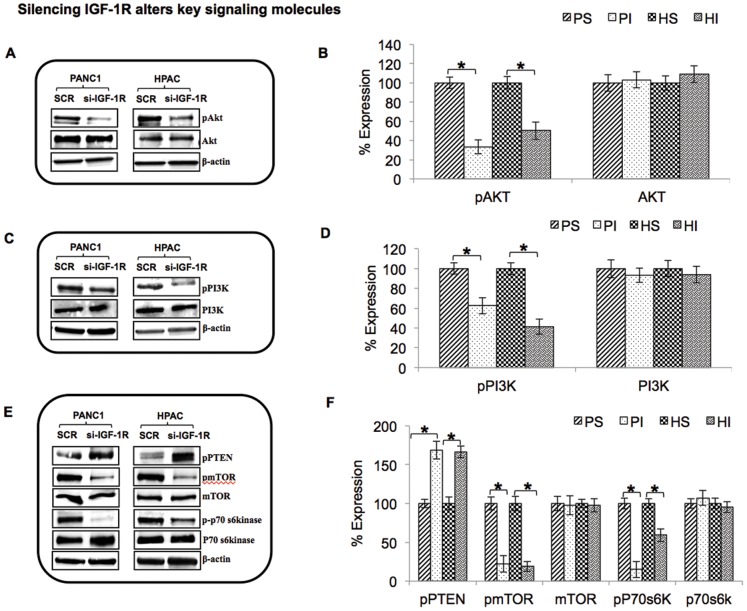
Suppression of IGF-1R alters key signaling molecules in PANC-1 and HPAC cells. (A, C & E): The effect of IGF-1R suppression on AKT/PI3K signaling was examined in pancreatic cancer cells. PANC-1 and HPAC cells were treated with IGF-1R siRNA for 48 h. The cells were harvested and the expression of phospho-AKT, AKT, phospho-PI3K, PI3K, phospho-PTEN, phospho-mTOR, mTOR, phospho-p70s6kinase, p70s6kinase and the internal control β-actin was measured by Western blotting. (B, D & F): Densitometric analysis is also shown to the right of each representative image.

Inhibiting only a single member of one particular signaling pathway or targeting only one particular cellular function may be insufficient to treat complex diseases like cancer. Therefore, targeting molecules with the most impact on multiple oncogenic signaling pathways will have greater translational potential for clinical PDAC treatment. In this regard, we chose to perform an even more comprehensive analysis of the effects of IGF-1R silencing in PANC-1 and HPAC cells and have verified that IGF-1R does in fact coordinate the regulation of multiple cellular pathways involved in survival, proliferation, metastasis, EMT, apoptosis and cell cycle signaling (Figure 5).

AKT is one of the most constitutively expressed molecules in many cancers and has been shown to enhance malignant cell proliferation [24]. We performed Western blot analysis in IGF-1R siRNA transfected cells in order to measure the expression levels of the AKT/PI3K signaling cascade, including active downstream and upstream effectors. We first measured the expression of active and total forms of AKT and found that the active form of AKT (pAKT) was significantly downregulated in IGF-1R silenced PANC-1 and HPAC cells while total AKT levels were unchanged (Figure 5A & B). A major upstream regulator of AKT is PI3K and IGF-1R suppression also inhibited the expression of pPI3K compared with scrambled control (Figure 5C & D).

Activation of P13K induces the phosphorylation of AKT which in turn activates mTOR and its downstream effector molecule p-p70s6kinase. Once again, IGF-1R silencing resulted in the suppression of the active forms of mTOR and p70s6k, while the total forms of the proteins remained unaltered (Figure 5E & F). Phosphatase and tensin homolog (PTEN), a potent inhibitor or negative regulator of PI3K, is mutated in most cancers at high frequency and associated with aggressive metastasis [25]. The loss of PTEN causes the dysregulation of PI3K, which could favor pancreatic carcinogenesis; however the comprehensive mechanism by which this occurs is not fully understood. PTEN was highly activated upon IGF-1R silencing in PANC-1 and HPAC cells (Figure 5E & F). Therefore, IGF-1R silencing induces PTEN expression and inhibits phosphorylation of AKT, PI3K, mTOR and p-70s6kinase in pancreatic cancer cells. These data suggest that silencing IGF-1R inhibits cell proliferation and enhances apoptosis by regulating activation status of PI3K/AKT pathway intermediates.

**Figure 6:**
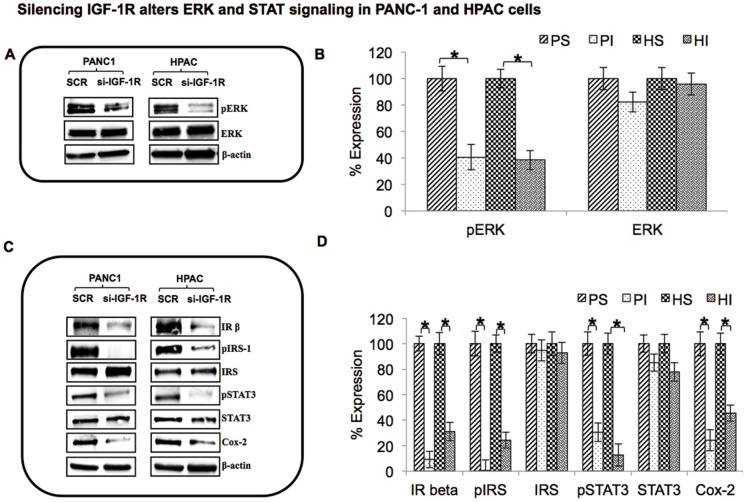
Silencing IGF-1R alters ERK and STAT signaling in PANC-1 and HPAC cells. (A & C): The effect of IGF-1R suppression on ERK and STAT signaling was examined in pancreatic cancer cells. Whole cell lysates were separated by SDS-PAGE and analyzed by Western blot for expression levels of phospho-ERK, ERK, IR-β, phospho-IRS-1, IRS, phospho-STAT3, STAT3, COX-2 and β-actin. (B & D): Representative blots are presented and corresponding densitometric analysis is shown to the right of each image. PS-PANC-1 Scrambled, PI-PANC-1 IGF-1R silenced, HS-HPAC Scrambled, HI-HPAC IGF-1R silenced.

ERK or Ras/Raf/MAP kinase signaling is one of the frequently up regulated pathways in most cancers including PDAC. This signaling pathway is known to regulate most cellular functions of the body such as survival, proliferation, mitosis, motility, differentiation, and apoptosis [24]. ERK is a highly attractive target for the treatment and development of anticancer drugs for human cancer. We therefore examined the effects of IGF-1R silencing on the expression of ERK. IGF-1R silencing resulted in effective inhibition of p-ERK, without any alterations in total ERK levels (Figure 6A & B). These results indicate that the ERK pathway also plays an important role downstream of IGF-1R in the proliferative and metastatic properties of pancreatic cancer.

The Janus kinase (JAK)/signal transducers and activators of transcription (STAT) pathway is aberrantly activated in malignant cancers. This signaling pathway is widely known for its role in proliferation, immune response and hematopoiesis in response to various growth factors and cytokines [26]. IGF-1R silencing profoundly decreased the levels of phosphoSTAT3, pro-inflammatory cytokine cyclo-oxygenease-2 (COX-2) and phospho-insulin receptor substrate-1(pIRS) (Figure 6C & D). Non-phospho specific forms of STAT3 and IRS remained unchanged in both scrambled control and IGF-1R silenced cells. Interestingly, targeting IGF-1R also inhibited the expression of Insulin receptor-β (IR-β) (Figure 6C & D).

All together, the results presented here demonstrate that knockdown of IGF-1R expression inhibits metastasis via inhibition of EMT. IGF-1R silencing also induces apoptosis and blocks cell proliferation via inhibition of PI3K/AKT, MEK/ERK, MAPK and JAK/STAT signaling. Our findings suggest that targeted therapy against IGF-1R would be effective and beneficial for treatment of patients with aggressive metastatic pancreatic ductal adenocarcinoma.

## Discussion

Our present study focuses on defining the role of IGF-1R in pancreatic cancer and lays a strong foundation for the deeper and broader understanding of the molecular mechanisms by which IGF-1R contributes to PDAC pathogenesis. This preliminary study is a springboard for discovering predictive biomarkers useful for PDAC diagnosis and therapy. IGF-1R is upregulated in a great proportion of cancer cells [27], [28]. We also found aberrantly over expressed IGF-1R in pancreatic cancer cell lines (PANC-1 and HPAC) and in human pancreatic adenocarcinoma tissues. Decreased proliferation and anchorage independent growth was observed upon effective IGF-1R silencing which indicates that there is a possibility of extending the life expectancy of PDAC patients through IGF-1R targeted therapies.

Epithelial-mesenchymal transition (EMT) is well-known as a crucial event during cancer invasion and metastasis and was also recently associated with poor disease prognosis in PDAC patients [29]. In a recent study, IGF-1R was shown to be a critical and important driver for EMT related events in lung cancer [30]. In fact, overexpression of IGF1R was found to be associated with increased mortality in most cancer patients due to enhanced potential for metastasis [31]. Similar to these prior results, when we knocked down IGF-1R, we observed reduced motility and migratory capabilities of PDAC cells. Thus the invasive properties of PDAC are significantly inhibited when IGF-1R is silenced.

Loss of E-cadherin, which is a hallmark of increased EMT, is sufficient to confer metastatic properties on breast cancer cells [23]. Our results demonstrate that silencing of IGF-1R inhibits metastasis of pancreatic cancer cells by enhancing the expression of E-cadherin. Our data further demonstrates that IGF-1R knockdown leads to the suppression of other key regulators of EMT such as notch-2, snail, zeb, slug, and the mesenchymal proteins N-cadherin and vimentin. These molecular alterations in response to IGF-1R knockdown could contribute significantly to the inhibition of EMT in pancreatic cancer cells. Thus inhibition of EMT is likely a primary factor contributing to reduced invasion and metastatic potential of pancreatic cells where IGF-1R is silenced. Targeting a master molecule like IGF-1R is therefore much more beneficial for a disease like PDAC than targeting only one or two components at a time.

Elevated levels of IGF-1 are known to play a pivotal role in regulating cell proliferation, differentiation and apoptosis via IGF-R signaling [32]. In cells highly dependent on IGF-1R signaling, apoptosis is highly induced simply by inhibiting IGF-1R expression [27]. However the role of IGF-1R signaling is not yet well studied in pancreatic cancer cells. Here, we observed the induction of apoptosis at a high frequency in IGF-1R silenced pancreatic cancer cells with increasing time of transfection. We found that inhibition of the IGF-1R signaling axis reduced the expression of the anti-apoptotic protein Bcl-2 and increased expression of pro apoptotic Bax. The classical initiator and effector molecules of apoptosis such as caspase8, caspase3 and cleaved PARP were upregulated in IGF-1R silenced cells indicating that apoptosis occurs via both death receptor and mitochondrial mediated pathways. Therefore, both intrinsic and extrinsic apoptotic pathways are effectively activated by IGF-1R silencing.

Interestingly, IGF1R inhibition by siRNA simultaneously reduced the levels of insulin receptor (IR) in both PDAC cell lines. Dual inhibition of IR and IGF-1R may confer an enhanced antitumor effect because IR signaling is also implicated in the pathogenesis of various tumors [33], [34]. IGF-1R-targeted inhibition is known to have certain metabolic consequences, which include elevation of blood glucose and insulin levels through feedback inhibition of the GH-IGF-1 signaling axis [32], [35]. IGF-1R blocking therapy, however, is reported to be well-tolerated in clinical phase I and II trials with only 3 to 25% of participants developing hyperglycemia [32], [35].

Signal transduction cascades downstream of both IGF-1R and IR were effectively inhibited by IGF-1R knockdown, including PI3K/AKT, MEK/ERK and JAK/STAT pathways. Hyperactivated AKT and PTEN loss have been reported in several cancers including pancreatic cancer and are associated with increased proliferation, metastasis, angiogenesis, cell growth and resistance to apoptosis [36]–[41]. IGF-1R silencing increases PTEN expression and negatively regulates the PI3K/AKT tyrosine kinase activities to enhance the anticancer effect in PDAC. Tumor suppressor PTEN was also reported to suppress the MAP kinase signaling via Shc phosphorylation [24] and IRS phosphorylation. Similarly, IGF-1R suppression inactivated the phospho IRS-1 expression levels thereby suppressing MAP kinase and PI3K/AKT signaling in PDAC cells. Extracellular signal-regulated kinase (ERK), the effector molecule of MAPK signaling, was strongly inhibited by silencing IGF-1R and this could have contributed to the decreased proliferation of PDAC cells. Currently mTOR is considered as one of the potential alternative targets for PDAC treatment and is also considered as a master regulator similar to what we show here for IGF-1R in pancreatic cancer [42]. It is therefore very encouraging that IGF-1R silencing strongly blocked the phosphorylation of mTOR and its downstream target p70S6Kinase through PI3K/AKT inhibition. Taken together, our findings support the notion that silencing IGF-1R signaling inhibits the downstream PI3K/AKT/MAPK pathways which also affects pathways even further downstream, such as mTOR/p70S6K.

Cytokines like TNF-α are the mediators that link inflammation and cancer [43], [44]. Activation of STAT3 and key inflammatory molecules such as nuclear factor kappa-B (NF-κB) induces COX-2 expression which in turn produces prostaglandins, resulting in upregulation of proinflammatory processes that enhance breast carcinogenesis [45], [46]. Previous studies also show that PI3K inhibitors blocked the phosphorylation of AKT and COX-2 expression to induce apoptosis in PTEN mutated human endometrial cancer cells [47]. Similar to this, we also found that AKT and ERK activation was blocked by IGF-1R siRNA in pancreatic cells leading to significant inhibition of the pro-inflammatory molecule COX-2. In a transgenic mouse model fed with high fat diet, the KRAS oncogene was activated downstream of COX-2 which mediated pancreatic inflammation that ultimately led to PDAC progression [48]. In our study, reduced COX-2 and pSTAT3 expression achieved by IGF-1R inhibition indicates the potential role of IGF-1R in inflammation mediated carcinogenesis of pancreatic cancer. In this regard, we believe there is a primary role for IGF-1R in inflammatory related pancreatic carcinogenesis.

## Conclusion

In summary, our study demonstrates that silencing IGF-1R strongly inhibits proliferation, colony forming capability, migration, and invasive/metastatic potential of pancreatic ductal adenocarcinoma cells. Further, we show that this occurs through induction of apoptosis and inhibition of EMT. Key molecular pathways affected by IGF-1R silencing in PDAC included PI3K/AKT, MAPK/ERK and JAK/STAT signaling cascades. We conclude that IGF-IR-targeting drugs hold much promise for the treatment of PDAC due to the nearly universal effects of IGF-1R in regulating various pathways involved in PDAC tumorigenesis. However, it remains important to ensure that the beneficial effects for PDAC treatment will not be outweighed by potentially harmful off-target physiologic effects. Therefore, our specific aim in our future work is to identify a molecular target that would be more specific for pancreatic cancer cells compared to normal cells which would still effectively target most of the protumorigenic functions of IGF-1R in PDAC.
